# Repair of Achilles sleeve avulsion: a new transosseous suture technique

**DOI:** 10.1186/s13018-020-01699-2

**Published:** 2020-06-17

**Authors:** Yu-ping Yang, Ding-yu Wang, Lin-wei Wei, Ning An, Li-yuan Tao, Chen Jiao, Qin-wei Guo, Yue-lin Hu

**Affiliations:** 1 Institute of Sports Medicine, Peking University Third Hospital, Beijing, 100191 People’s Republic of China; 2 Pharmacy Department, Peking University Third Hospital, Beijing, 100191 People’s Republic of China; 3 Research Center of Clinical Epidemiology, Peking University Third Hospital, Beijing, 100191 People’s Republic of China

**Keywords:** Sleeve avulsion, Achilles tendinopathy, Suture, Transosseous, Achilles tendon

## Abstract

**Background:**

Achilles sleeve avulsion usually occurs from pre-existing insertional Achilles tendinopathy, leaving a calcific spur at the insertional site. The purpose of this study was to introduce a new technique using the spur base on the insertional site to drill the suture tunnel to repair Achilles sleeve avulsion.

**Methods:**

In total, 11 patients diagnosed with Achilles sleeve avulsion underwent this new surgical technique and were followed for a mean time of 40 months. Clinical outcomes were measured using the visual analog scale (VAS), American Orthopaedic Foot and Ankle Society (AOFAS) score, Victorian Institute of Sports Assessment-Achilles (VISA-A) score, Tegner score, and time taken to return to activities. Preoperative and postoperative MRI, the ability to perform heel rise, and complications were also evaluated.

**Results:**

All cases (11/11) had insertional Achilles tendinopathy with calcific spur formation on the tendon’s insertion. At final follow-up, the average VAS score improved from 5.3 to 0.1, AOFAS score improved from 44.8 to 97.9, VISA-A score improved from 23.6 to 96.6, and Tegner score improved from 0.9 to 4.9. Tendinopathy symptoms were eliminated. Patients returned to daily activities, work, and sports 3.5 months, 2.8 months, and 12.3months after operation, respectively. Patients took an average of 18.1 weeks after operation to perform the single heel rise test. No severe complications such as infection and rerupture were observed.

**Conclusion:**

Our new transosseous suture technique is a promising alternative option in treating Achilles sleeve avulsion. More cases and longer follow up are needed in order to find the best reconstructive option for this pathology.

**Levels of evidence:**

Level IV

## Introduction

The Achilles tendon is the largest and the most frequently ruptured tendon in the lower extremity. The most frequent site of rupture is approximately 2 to 6 cm proximal to the insertion of the tendon on the calcaneus, whereas more distal ruptures occurring at the calcaneal insertion—also known as Achilles sleeve avulsion—are less common [1]. In most cases, Achilles sleeve avulsion occurs from insertional Achilles tendinopathy and is accompanied by bone spurs and calcification formation in the tendon at the insertion site [2]. In addition, tendon degeneration, calcific spur formation, and small transverse tears at the tendon–bone junction may jeopardize the strength of the Achilles tendons [3–5]. A recent study of 12 professional athletes and 16 patients from the general population determined the presence of retrocalcaneal bursitis, edema within the Achilles insertional sleeve, and calcaneal edema in the prerupture state [6]. Patients usually complain of prodromal pain and stiffness on arising after sleep or after sitting for a prolonged time. When a sudden force or continually increasing tension and strength is applied to the Achilles tendon insertion, Achilles sleeve avulsion occurs, leaving a calcific spur—usually visible on a lateral ankle radiograph—at the insertional site and the calcified tendon end.

Although conservative treatment with dynamic rehabilitation in acute Achilles tendon rupture has achieved promising outcomes, no successful use of conservative treatment for Achilles sleeve avulsion has been reported [7]. The tendon–bone junction is slow to heal because of the relative avascularity of the fibrocartilage zone and bone loss at the site of injury. Moreover, the structure and composition of the native direct tendon–bone interface is not reformed during healing, increasing the risk for failure of the tendon attachment [8, 9]. Additionally, the rupture end usually contains degenerative tendon tissue and calcification. Therefore, operative treatment for Achilles sleeve avulsion is often a better choice [6]. However, the repair of Achilles sleeve avulsion is challenging for surgeons because it leaves very little tissue for direct repair of the Achilles tendon onto its insertion at the calcaneus. Open reduction and screw fixation, usually used to fix large bone fragment, cannot effectively resist the pull-out tension of the triceps surae [10]; Achilles sleeve avulsion fixation has no existing standard operative operation. The current operative technique includes suture and anchor, transcalcaneal suture, or some combination of both [11–16]. Although good to excellent outcomes have been reported, these techniques have drawbacks such as high-cost foreign material implantation and infection risk in the case of the suture anchor technique [13, 15] and technically demanding operation and extensive iatrogenic injury in the case of the transcalcaneal suture technique [11, 16].

By debriding the degenerative tendon tissue and calcified spur, tendinopathy problems can be eliminated. The base of the spur also offers a stump for tunnel drilling and suture fixation of the Achilles tendon. The purpose of this study was to introduce a new technique by using the spur base on the insertional site to drill the suture tunnel and repair Achilles sleeve avulsion, thus providing an alternative option for treating Achilles sleeve avulsion.

## Materials and methods

The study protocol was approved by the Ethics Committee of Peking University Third Hospital (IRB00006761-M2018008). All patients read and signed a written informed consent form. All methods in this study were performed in accordance with relevant guidelines and regulations. We identified 12 patients with Achilles sleeve avulsion from November 2013 to March 2016. All patients underwent a surgical procedure performed by one of the authors. Achilles sleeve avulsion was diagnosed using by clinical presentations and physical examination, routine ankle X ray and MRI. Exclusion criteria were calcaneal tuberosity avulsion fractures—type I or II fracture of the calcaneus tuberosity, as described by Beavis (2008)—non-insertional Achilles rupture, Achilles tendon rerupture, and previous surgical procedure on the affected Achilles tendon [17].

One patient was lost to follow-up because she could not be contacted, having moved out of the country. The remaining 11 patients (10 men and one woman, average age 46years) were reviewed with an average follow-up time of 40 months (30–57 months). Two patients had raised total cholesterol. Three patients had raised low-density lipoprotein-cholesterol. None of the patients was using statin before the surgery. Patient demographic information, mechanism of injury, prodromal Achilles tendon disorders (including Haglund’s deformity and insertional Achilles tendinopathy), and time from injury to operation are listed in Table 1. All patients in our series met the East Asian criteria of overweight or obesity with a mean body mass index of 26.5 kg/m^2^. All cases (11 of 11) had insertional Achilles tendinopathy with calcific spurs on the tendon’s insertion. No patient had a history of Achilles insertional corticosteroid injection prior to rupture.

**Table 1:** Patient demographics

Case	Age	Gender	BMI	Injured side	Follow-up time (month)	Mechanism of injury	Haglund’s deformity	Insertional Achilles tendinopathy	Time before operation (day)
1	34	M	26.0	R	57	Soccer	Yes	Yes	2
2	39	M	27.8	L	47	Soccer	No	Yes	3
3	57	M	26.1	R	42	Falling down	No	Yes	5
4	35	M	28.4	R	41	Basketball	No	Yes	2
5	46	M	26.8	R	41	Badminton	Yes	Yes	2
6	63	M	24.8	R	41	Stepping in a hole	No	Yes	6
7	51	F	28.6	L	39	Ankle sprain	Yes	Yes	5
8	52	M	26.1	R	36	Basketball	Yes	Yes	7
9	38	M	24.1	R	32	Walking down the stairs	No	Yes	2
10	67	M	26.8	L	31	Falling down	Yes	Yes	13
11	30	M	25.7	L	30	Soccer	No	Yes	11

*M* male, *F* female, *L* left, *R* right

### Clinical evaluation

Subjective outcomes were American Orthopaedic Foot and Ankle Society (AOFAS) score, visual analog scale (VAS), Victorian Institute of Sports Assessment-Achilles (VISA-A) score, Tegner score, and time taken to return to activities. Outcomes were evaluated by two authors according to the study questionnaire at five time points: before the operation; 3, 6, and 12 months after the operation; and September 2018 (in a telephone follow-up). Because the preserved spur base was part of tendinopathy, we paid special attention in the telephone follow-up to remaining tendinopathy symptoms such as pain and stiffness on the heel. The ability to perform heel rise on both the limbs and the injured limb was evaluated by one of the authors during the patient’s return visit. Patients were asked to perform 10 single-limb heel rises on either both or only the affected side and were assessed as being able or unable to do so [18]. Hyperdorsiflexion sign was also examined [19]. All patients underwent MRI for an evaluation of the contour and signal intensity of the Achilles tendon preoperatively and at either 3 or 6 months postoperatively. Two radiologists independently assessed MRI results.

### Operative technique

**Fig. 1:**
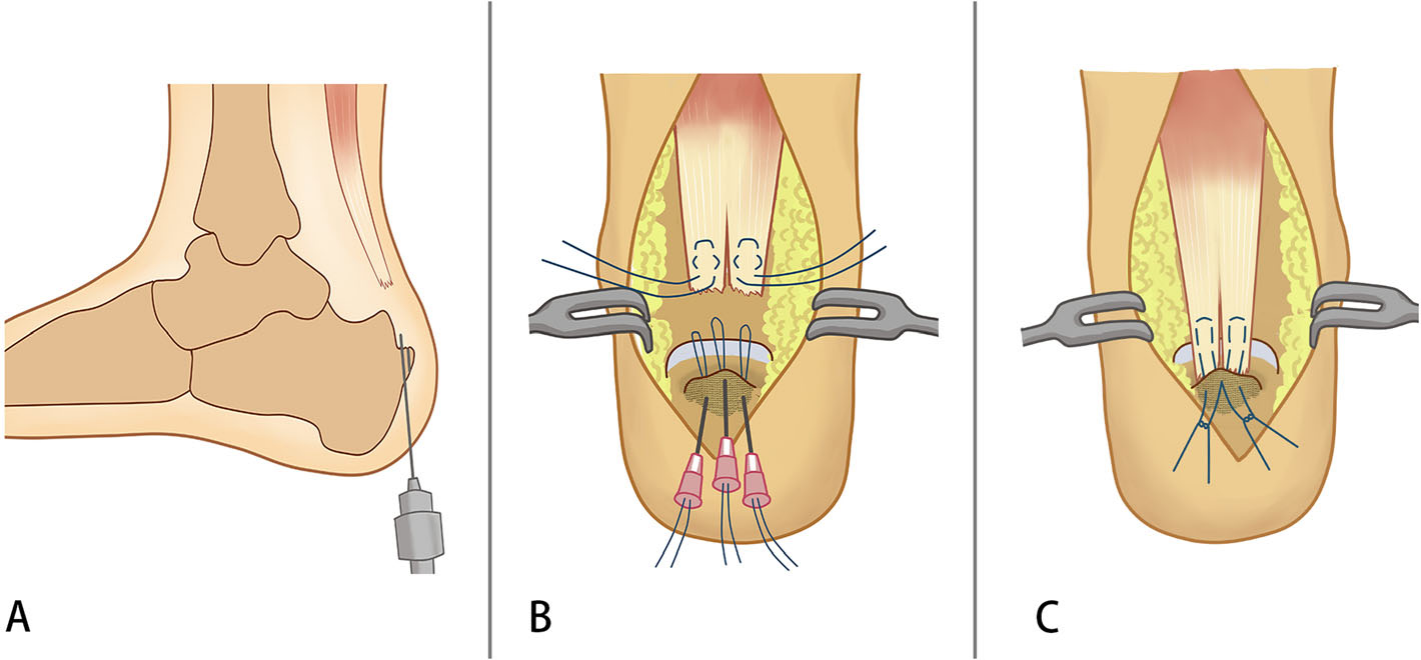
Illustrations of the operative technique. **a** Avulsed bone fragment was removed from the tendon, and the Achilles tendon end was debrided. Three bone tunnels arranged in a triangle shape were drilled using a 1.5-mm Kirschner wire through the bone spurs base. **b** The Achilles tendon was woven with two sutures. Three 12 G syringe needles with 4-0 thread loop passed tunnels to pull the four free ends of sutures through the tunnels. The Achilles tendon end was reattached to the insertional site. **c** The Achilles tendon was pulled back down to the insertional site

**Fig. 2:**
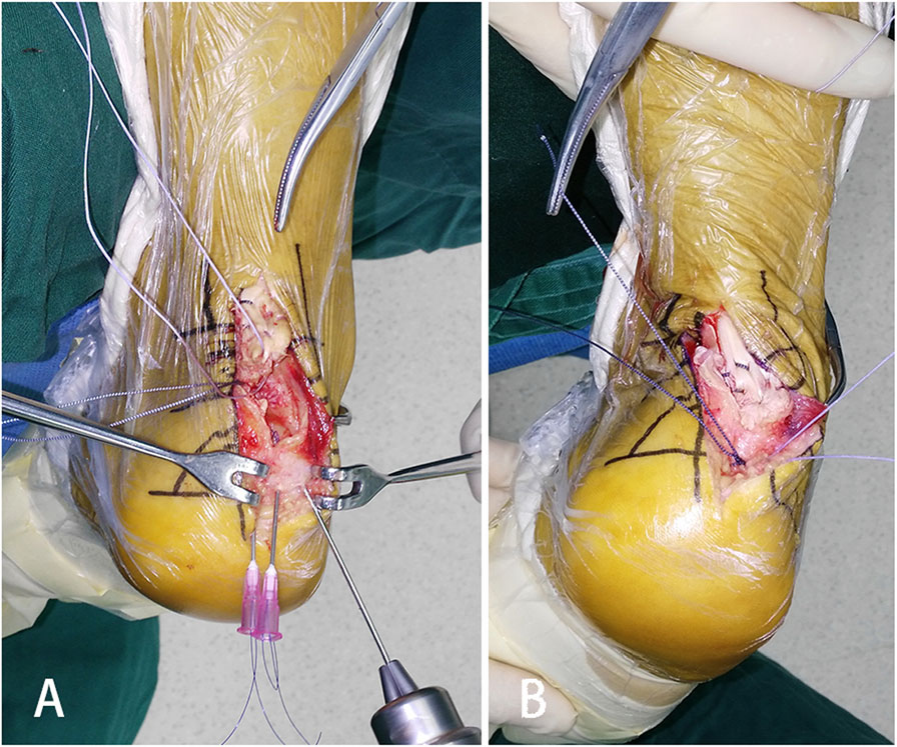
Operative photograph of fixation technique. **a** A 1.5-mm Kirschner wire was used to drill three bone tunnels through the bone spurs base and syringe needles carrying thread loop passed tunnels to help pull the suture ends through the tunnels. **b** The tendon end was pulled back and fixed onto the insertional site using sutures

Patients were operated on in the prone position under lumbar anesthesia with a thigh tourniquet. Skin preparation was performed, and sterile drapes were applied. A medial incision was made along the Achilles tendon, which allowed for visualization of the calcaneal tuberosity and avulsed bone fragment. The avulsed bony fragment was removed from the tendon, and the degenerative tissue was debrided. Two whip stitches using No. 2 Orthocord sutures (DePuy Mitek, USA) were attached to the medial and lateral side of the Achilles tendon, leaving four free ends. The Haglund’s deformity was resected if cartilage lesions were present on the Haglund exotosis. The main body of bone spurs at the insertion site was removed, and an approximately 5-mm-thick flat base of the bone spur was reserved. A 1.5-mm Kirschner wire was used to drill three bone tunnels that were arranged in a triangle shape through the bone spur base (Figs. 1a and 2a). To facilitate the firm tying of suture ends, a nucleus pulposus clamp was used to make grooves between the holes. Subsequently, three 12 G syringe needles (WEGO, Shandong, China) with a 4-0 thread loop (Securex, Spain) passed the tunnels, and four free ends of sutures were pulled through the tunnels (Figs. 1b, 2a). The first central tunnel held two free ends from different sutures, and each tunnel in the lateral held the other free end of sutures. The Achilles tendon was pulled back down to the insertional site, and the distal end of the Achilles tendon reached the flat base surface. Plantar flexion was the same for the uninjured side in the resting position. The suture ends were then tied together (Fig. 2b), and the wound was closed in layers. The mean operation time was 73 min.

### Postoperative rehabilitation

Postoperatively, patients wore a below-the-knee cast that held the ankle in a slight plantarflexion for 8 weeks. A rehabilitation program was initiated shortly after the operation. Patients were encouraged to perform active flexion and extension of the hallux and toes, isometric exercises of the quadriceps muscles 500 times a day, straight-leg raises 30 to 50 times a day, and active flexion and extension of the knee while in their short leg cast. Six weeks after surgery, patients were allowed to remove the short leg cast but only to perform active plantar flexion and dorsiflexion of the ankle. Patients performed weight bear and morning inversion and eversion exercises while wearing their cast. Ideally, the ankle exercise should not cause pain or be done passively. After the cast was removed, patients were allowed to walk with the help of elbow crutches while wearing boots that had a 3.0- to 3.5-cm heel lift. The heel lift was cut in half after 6 weeks and removed after another 6 weeks. Elbow crutches were gradually removed over 4 weeks. Patients were encouraged to gradually return to playing sports and engaging in daily activities.

### Statistical analysis

SPSS 22 software was used for statistical analysis. Paired Student’s *t* test was used to analyze continuous variables. A two-tailed *p* value of < 0.05 was considered statistically significant.

## Results

Patients reported the elimination of all preinjury tendinopathy symptoms. The mean VAS score for pain decreased from a preoperative score of 5.3 to the 3-month, 6-month, and 1-year postoperative scores of 1.7, 0.6, and 0.2, respectively. The AOFAS Ankle-Hindfoot score improved from a preoperative score of 44.8 to the 3-month, 6-month, and 1-year postoperative scores of 83.2, 91.1, and 94.3, respectively. The VISA-A score also improved from a preoperative score of 23.6 to the 3-month, 6-month, and 1-year postoperative scores of 73.1, 83.7, and 83.9, respectively. Likewise, the Tegner score improved from a preoperative score of 0.9 to the 3-month, 6-month, and 1-year postoperative scores of 2.4, 3.5, and 4.4, respectively. At final follow-up, the average VAS, AOFAS, VISA-A, and Tegner scores were 0.1, 97.9, 96.6, and 4.9, respectively (Table 2). Patients returned to daily activities, work, and sports 5 months, 2.8 months, and 12.3 months after operation, respectively. Patients could perform heel rise on both the limbs and the operative limb 13.5 weeks and 18.1 weeks after operation, respectively (Table 3). The hyperdorsiflexion sign was negative in all patients. At the final follow-up, 10 of 11 patients could play sports using ankle for more than 30 min, performed 10 single heel rises and 10 single leg hops without pain. One patient still had limited ankle flexion and extension, and mild pain during sports and 10 single heel rises examination.

**Table 2:** Preoperative and postoperative final follow-up results of VAS, AOFAS, VISA-A, and Tegner score. Values were reported as mean ± SD

	Preoperative	Postoperative	*P* value
VAS	5.3 ± 2.8	0.1 ± 0.3	< 0.05
AOFAS	44.8 ± 23.4	97.9 ± 5.8	< 0.05
VISA-A	23.6 ± 16.5	96.6 ± 6.1	< 0.05
Tegner	0.9 ± 0.8	4.9 ± 1.3	< 0.05

**Table 3:** Patients postoperative functional outcomes. Values were reported as mean ± SD

Heel rise on both limbs (week)	Heel rise on the operative limb (week)	Return to daily activities (month)	Return to work (month(s))	Return to sports (month)
13.5 ± 4.9	18.1 ± 7.4	3.5 ± 1.3	2.8 ± 1.3	12.3 ± 0.5

Thrombophlebitis, delayed wound healing, infection, and rerupture did not occur in any patient. Two patients had numb skin around their heels after the operation, but they recovered—one after 6 months and another after 30 months. Ten patients had postoperative ankle swelling, and six recovered after 3 months, two recovered after 6 months, and two recovered after 1 year.

**Fig. 3:**
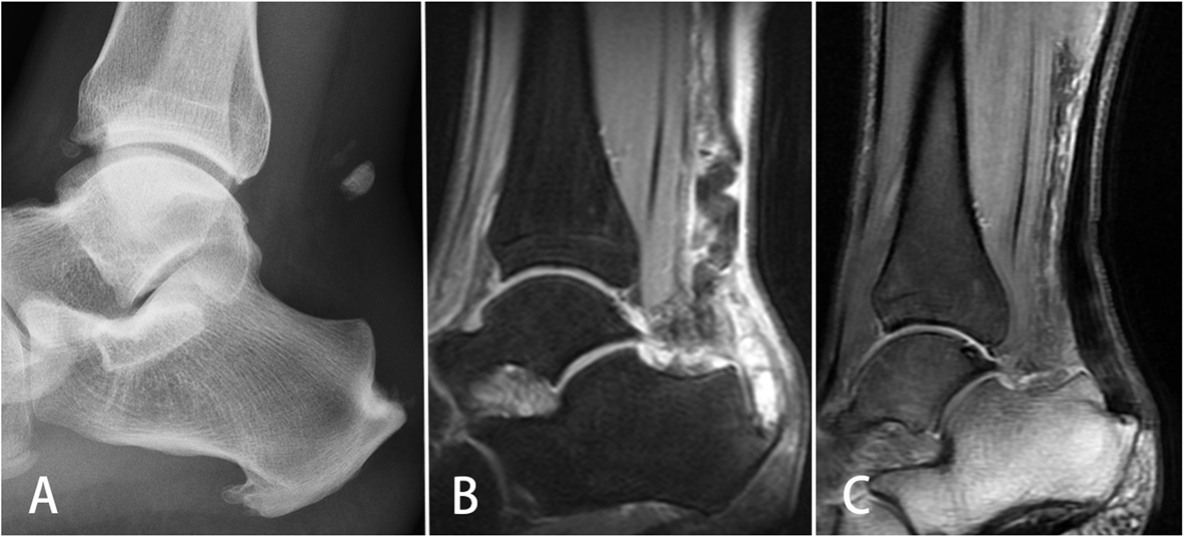
Preoperative and postoperative radiograph and MRI image of a patient (case 1). **a** Preoperative lateral ankle radiograph of a 34-year-old man who sustained a distal Achilles tendon rupture on his right lower limb 2 days before. An avulsed bone fragment was present with a 5.1-cm shift distance. Calcific spur formation was present at the tendon insertional site. **b** Preoperative T2-weighted MRI of the ankle demonstrated Achilles tendon rupture and retraction. **c** Six months after operation, T2-weighted MRI of the ankle demonstrated that Achilles tendon was attached to the calcaneus and the distal part of the Achilles tendon was enlarged

Preoperative MRI images for all cases indicated a discontinuity of the Achilles tendon and a bony fragment in the tendon end (Fig. 3a). The average Achilles tendon shift distance was 4.6 cm (3.4 to 6.3 cm). All the patients had soft tissue swelling around their ankle. Five patients had ankle joint effusion. Three patients had anterior talofibular ligament and calcaneofibular ligament injury. One patient had osteochondral lesion of the talus. Two patients had calcaneal edema and retrocalcaneal bursitis. Postoperative MRI indicated a normal continuity of the Achilles tendon for all cases (Fig. 3b and c). Four patients had Achilles tendon thickening. A partial discontinuous fibrous bundle was observed in one patient.

## Discussion

Compared with the more common Achilles rupture, Achilles sleeve avulsion is much rarer. A previous study reported that 7.6% of all operatively managed Achilles ruptures were distal ruptures [12]. According to unpublished data from our institution collected over the previous 5 years, Achilles sleeve avulsion only constituted 2.6% of all Achilles tendon ruptures. A previous study reported that Achilles sleeve avulsion typically arose from insertional Achilles tendinopathy [11, 12]. We also observed a high prevalence (11 of 11 cases) of pre-existing insertional Achilles tendinopathy in our series. Tendinopathy weakens the strength of the Achilles tendon at the insertional site, and when a sudden force is applied to the Achilles tendon, rupture occurs. Because obesity increases the Achilles tendon load, Holmes reported a significant correlation between obesity and Achilles tendinopathy [20]. All 11 patients in our series met the criterion of being overweight or obese. Similar findings were reported by Huh [12]. To reduce the risk of Achilles sleeve avulsion, we suggest weight loss and health education for overweight patients who have presented tendinopathy symptoms.

Achilles sleeve avulsion has no standard operative technique. Current operative techniques can be distinguished into suture anchor fixation techniques, transcalcaneal drilling techniques, and some combination of both [11–16, 21, 22]. The suture anchor technique is more accepted by surgeons. Without a plantar incision, suture anchor fixation provides a less invasive option relative to the transcalcaneal tunnel technique. However, metal implantation increases the infection risk, causing surgical failure [23]. The cost of implantation is also higher. The more technically demanding transcalcaneal bone tunnel technique may likewise increase the risk of calcaneal stress fracture.

**Fig. 4:**
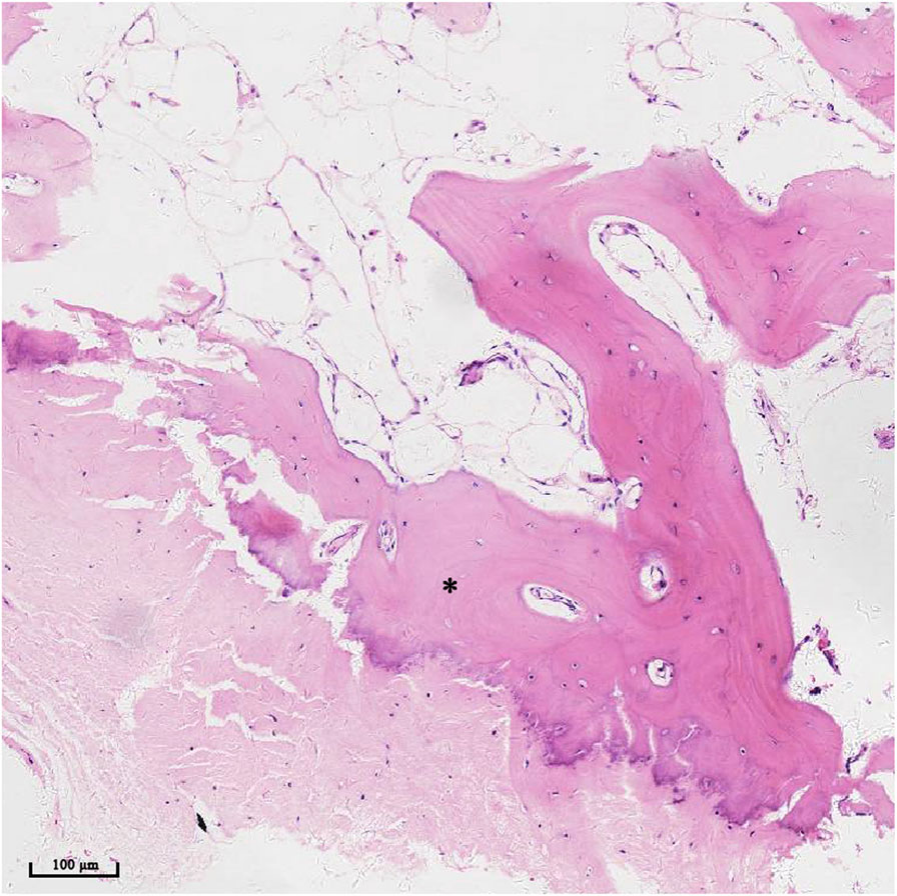
Pathological section of the bone-tendon interface from one patient. The bone tissue (*) where tendon attached is nearly normal. Thus, after the debridement, the flat bone base can be used to reconstruct the tendon

In this study, we found that all ruptures occurred at the middle part of the calcific spur, leaving a spur base at the calcaneus. Similar radiological features have been observed in other studies [12–14, 22]. Because the calcific spur is part of the usually painful problem of Achilles tendinopathy, we removed degenerative tissue together with the main body of the calcified spur and only preserved an approximately 5-mm-flat base at the insertional site as the stump for the bone tunnels. The pathological sections have shown that the bone tissue where tendon attached is nearly normal (Fig. 4). At the final follow-up, VAS was 0.1 and patients reported no tendinopathy symptoms, suggesting that they recovered from Achilles tendinopathy problems. Reruptures were absent, and patients could perform single heel rise within 18 months, thus demonstrating that the base of the bone spur offered sufficient fixation strength. The operative area was restricted to the posteromedial aspect of the calcaneus, and we avoided drilling any holes through the calcaneus and plantar incision. Only surgical sutures and no implants were used to immobilize the distal Achilles tendon, thus avoiding foreign body implantation and reducing infection risk. Our surgery cost was also lower than that of suture anchor fixation. However, we have to admit that it is an open method and a high level of technical skill and experience are required to perform such operations.

It has been reported in the literature that different operative techniques have achieved satisfactory outcomes. Manisalco used three suture anchors and reported good to excellent outcomes in seven patients with a 6-month follow-up [15]. In a case series analyzed by Kilicoglu, four patients were treated with two suture anchors and had a reported AOFAS score of 88.75 [13]. Although the dorsiflexion of the injured joints during the gait cycle was significantly less than that of the normal sides, they detected no obvious differences between the kinematic gait parameters of the injured and normal ankles. Huh used two suture anchors and reported a 92.8 AOFAS score for 11 patients [12]. Bibbo used transcalcaneal drilling in six patients and reported no statistically significant difference between the AOFAS score of the operative and unaffected limb [11]. In a case series analyzed by Schipper that included 16 patients from the general population and 12 professional athletes with a median follow-up of 8.1 years, high patient satisfaction and good clinical outcomes were reported; all athletes returned to play, without re-rupture, after an average of 13.4 months [6]. Maffulli presented a novel surgical technique using a free ipsilateral semitendinosus graft and interference screw fixation and reported good or excellent results in 28 patients [24]. In our study, all patients could perform a single heel rise test on the injured side at an average of 18.1 weeks after the operation. Patients also resumed sports and their daily activities at an average of 3 months and 1 year, respectively. The mean AOFAS score in the final follow-up was also 97.9. No severe complications such as re-rupture and infection were reported in the 40-month follow-up, suggesting that our technique was safe and reliable. In our surgery, a medial incision was made and care was taken to minimize nerve injury. When operating on two patients, we felt a higher tension on the tendon when tying it to the calcaneus. However, no visible Achilles tendon elongation was noted in the postoperative examination of the hyperdorsiflexion sign, and the two patients exhibited satisfactory outcomes.

Postoperative management is as important as a successful surgery. Patients should wear a leg cast for at least 6 weeks. Most importantly, care should be taken to avoid falls, which can cause re-rupture. Weight-bearing walking in boots that have a 3.0- to 3.5-cm heel lift may reduce the likelihood of Achilles tendon elongation and facilitate excellent functional outcomes. Notably, patients with delayed recovery, ankle swelling, or long-lasting pain were usually patients who did not actively follow the rehabilitation program. The fear of pain and re-rupture was the primary cause of poor compliance. Thus, doctor supervision and encouragement are necessary during rehabilitation.

Our study has several limitations. Although our study, at 11 patients, has the second-largest series of Achilles sleeve avulsion in the literature, because of its retrospective design, absence of a control group, and small sample size, we cannot conclusively determine the superiority of our new technique over its counterparts. Moreover, our surgical technique is inapplicable in cases where the whole calcific spur is avulsed. In our study, all rupture sites occurred at the middle of the spur, leaving a 4.5- to 17.2-mm stump at the insertional site. Radiograph and MRI findings in other studies have also indicated the existence of an insertional spur. Thus, our technique is applicable in most cases (where the spur remains at the insertional site) [6, 12, 14]. The studied group is heterogeneous in terms of both gender and age for the Achilles sleeve avulsion is a rare condition. The VAS, AOFAS, VISA-A, and Tegner scores are also self-reported instruments and thus subject to bias. However, because these scales have been used in other studies, we had to use these scales for comparison between our outcomes and those in the literature. No other objective functional test than single-limb heel rise was took to examine functional recovery.

## Conclusion

Our new transosseous suture technique is a promising alternative option in treating Achilles sleeve avulsion. More cases and longer follow-up are needed in order to find the best reconstructive option for this pathology.

## Data Availability

All data generated or analyzed during this study are included in this published article.

## Abbreviations

VAS: Visual analog scale
AOFAS: American Orthopaedic Foot and Ankle Society
VISA-A: Victorian Institute of Sports Assessment-Achilles
